# Selective loss of Purkinje cells in a patient with anti-gliadin-antibody-positive autoimmune cerebellar ataxia

**DOI:** 10.1186/1746-1596-6-14

**Published:** 2011-02-04

**Authors:** Kazunori Nanri, Makoto Shibuya, Takeshi Taguchi, Akira Hasegawa, Nobuyuki Tanaka

**Affiliations:** 1 Department of Neurology, Tokyo Medical University Hachioji Medical Center 1163 Tatemachi, Hachioji, Tokyo, 193-0998, Japan; 2 Department of Pathology, Tokyo Medical University Ibaraki Medical Center, Ibaraki, Tokyo, Japan

## Abstract

The patient was an 84-year-old woman who had the onset of truncal ataxia at age 77 and a history of Basedow's disease. Her ataxic gait gradually deteriorated. She could not walk without support at age 81 and she was admitted to our hospital at age 83. Gaze-evoked nystagmus and dysarthria were observed. Mild ataxia was observed in all limbs. Her deep tendon reflex and sense of position were normal. IgA anti-gliadin antibody, IgG anti-gliadin antibody, anti-SS-A/Ro antibody, anti-SS-B/La antibody and anti-TPO antibody were positive. A conventional brain MRI did not show obvious cerebellar atrophy. However, MRI voxel based morphometry (VBM) and SPECT-eZIS revealed cortical cerebellar atrophy and reduced cerebellar blood flow. IVIg treatment was performed and was moderately effective. After her death at age 85, the patient was autopsied. Neuropathological findings were as follows: selective loss of Purkinje cells; no apparent degenerative change in the efferent pathways, such as the dentate nuclei or vestibular nuclei; no prominent inflammatory reaction. From these findings, we diagnosed this case as autoimmune cerebellar atrophy associated with gluten ataxia. All 3 autopsies previously reported on gluten ataxia have noted infiltration of inflammatory cells in the cerebellum.

In this case, we postulated that the infiltration of inflammatory cells was not found because the patient's condition was based on humoral immunity. The clinical conditions of gluten ataxia have not yet been properly elucidated, but are expected to be revealed as the number of autopsied cases increases.

## Background

It has recently been reported that autoimmune cerebellar ataxias, such as gluten ataxia [1] and anti-glutamic acid decarboxylase (GAD)-antibody-positive cerebellar ataxia [2-4], are treatable. However, because of the small number of previous autopsy reports, the neuropathology and clinical conditions of autoimmune cerebellar ataxia are yet to be determined.

We experienced the case of an elderly woman who was suspected of autoimmune cerebellar ataxia associated with gluten ataxia due to the presence of IgG and IgA anti-gliadin antibody positivity and a positive response to high-dose immunoglobulin therapy. However, it was difficult to diagnose whether she had cerebellar atrophy or not.

The autopsy after her death at 85 showed selective loss of Purkinje cells and a diagnosis of autoimmune cerebellar atrophy was confirmed. However, the pathological findings differed to previous reports of gluten ataxia. Thus we present our own report with consideration of the clinical features.

## Case Presentation

**Figure 1:**
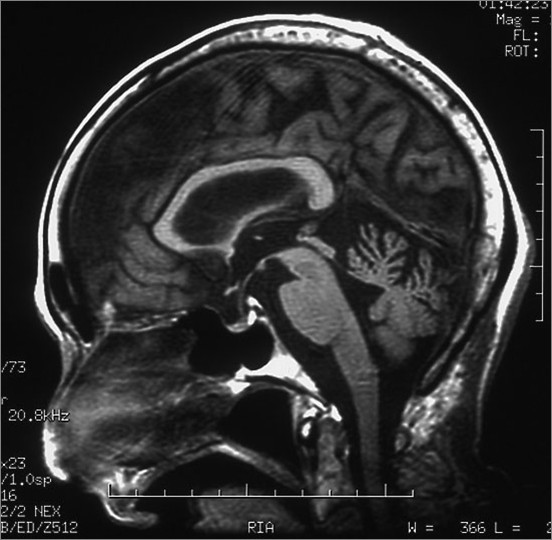
**Brain MRI**. Conventional brain MRI showed mild cerebellar atrophy, which seemed to be consistent with age.

**Figure 2:**
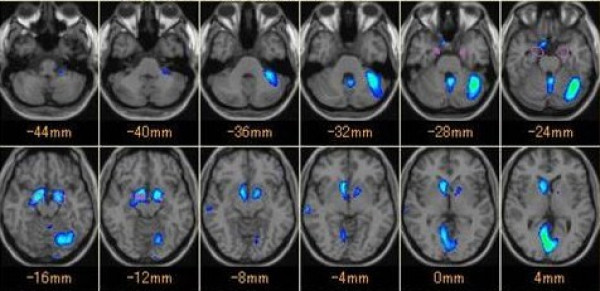
**MRI voxel based morphometry**. MRI voxel based morphometry revealed cortical cerebellar atrophy, which was left hemisphere dominant.

**Figure 3:**
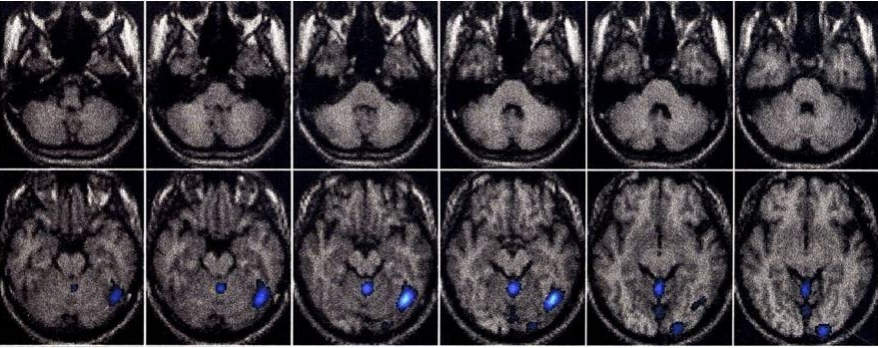
**SPECT-eZIS**. SPECT-eZIS revealed reduced cerebellar blood flow, which was left hemisphere dominant.

The patient was an 84-year-old woman who had the onset of truncal ataxia at age 77 and had a history of Basedow's disease. There was nothing significant in her family history. Her ataxic gait gradually deteriorated. At age 81, she could not walk without support. At age 83, she was admitted to our hospital. Gaze-evoked nystagmus and dysarthria were observed. The patient showed a wide-based gait and she required assistance to walk. Mild ataxia was observed in all limbs. Her deep tendon reflex and sense of position were normal. Her antibody levels were as follows: rheumatoid factor, 21 IU/mL (normal < 18 IU/mL); anti-SS-A/Ro antibody, >500 U/mL (normal < 10 U/mL); anti-SS-B/La antibody, 41.1 U/mL (normal < 10 U/mL); anti-TPO antibody, 1.0 U/mL; IgA anti-gliadin antibody, 42.7 EU (normal < 20 EU); and IgG anti-gliadin antibody, 21.9 EU (normal < 20 EU). Anti-Hu, anti-Yo and anti-GAD antibodies were all negative. A conventional brain MRI showed mild cerebellar atrophy, which seemed to be consistent with age (Figure 1). However, MRI voxel based morphometry (VBM) and SPECT-eZIS revealed cortical cerebellar atrophy and reduced cerebellar blood flow (Figure 2, Figure 3). A nerve conduction test was within the normal range. Cerebrospinal fluid examination showed a normal cell count, and the protein concentration was 40 mg/dL.

IVIg treatments were performed twice with an interval of 6 months between them, and her ICARS score improved from 31 to 22 at the first therapy and from 33 to 23 at the second therapy, indicating that IVIg therapy was moderately effective. After the IVIg treatment, the anti-TPO antibody level became negative, the anti-SS-A/Ro antibody level decreased to 391 U/mL, and the anti-SS-B/La antibody level decreased to 7.3 U/mL. The IgA anti-gliadin antibody level decreased to 3.7 EU. The patient died in her nursing home at age 85. The cause of death was not clear, but aspiration pneumonia was suspected and an autopsy was performed.

The autopsy was performed 24-hours after her death, and a localized lung abscess with Aspergillus infection was found. Marked atrophy of the thyroid gland was discerned, chronic inflammatory cell infiltration or follicular epithelial changes were not seen, and hyperplastic findings were not obvious. Severe atrophic changes in the salivary glands with lymphoid cell infiltration were also seen in association with Sjögren's syndrome. The digestive system exhibited severe post-mortem autolysis. Although precise histological examination could not be made, flattening, blunting, atrophy, disappearance or other findings related to the celiac disease were not obvious. In addition, benign or malignant tumors were not found.

A proportion of the cerebrum appeared to have been preserved. No prominent atrophic changes were identified and this included the hippocampus. Minimal cerebellar atrophy might have been present, but no atrophic change was discerned in the brain stem.

**Figure 4:**
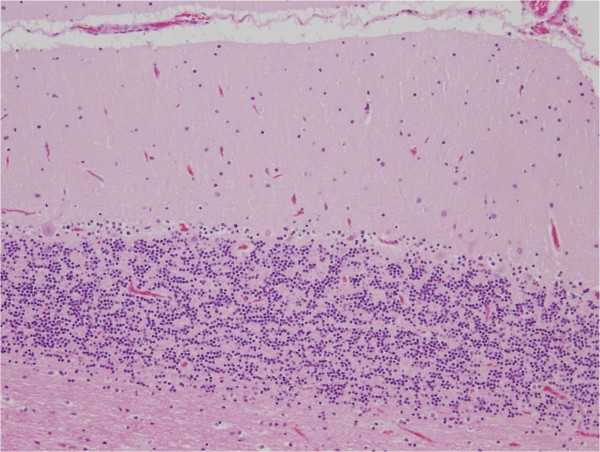
**Selective loss of Purkinje cells**. Histologically, the thickness of the molecular layer of the cerebellum appears to be preserved, but a mild thinning of the granular layers was observed. A mild to moderate decrease in the number of the Purkinje cells was observed in association with mild Bergman gliosis.

Histologically, the thickness of the molecular layer of the cerebellum appeared to have been preserved, but a mild thinning of the granular layers was observed. A mild to moderate decrease in the number of Purkinje cells was observed in association with mild Bergman gliosis elsewhere in the cerebellar cortex (Figure 4). Mild accentuation in the vermis and in the bottom of the cerebellar folia was noted. Empty basket cells were occasionally seen. Edematous splitting of the Purkinje cell layers was also noted. No apparent inflammatory cell reaction was identified after immunostaining for CD3, CD4, CD8, CD20, CD68 or CD79a. The dentate nucleus was preserved without apparent neuronal loss or gliosis, as were the inferior olivary nuclei and the vestibular nerve nuclei. The posterior column of the spinal cord was also preserved.

To summarize the neuropathological findings, selective loss of the Purkinje cells was found and there was no apparent degenerative change in the efferent pathways, such as the dentate nuclei or the vestibular nuclei. No prominent inflammatory reaction was identified.

## Discussion

In this patient, the culprit lesion of truncal ataxia was not determined until 7 years after onset of the diseases. During the 7 intervening years, neither clinical nor neurophysiological examinations had demonstrated any findings indicative of peripheral nerve disorder, and conventional MRI had also provided no distinct finding of cerebellar atrophy. Seven years after onset of the disease, SPECT revealed slight reduction in blood flow in the cerebellum, which was left hemisphere dominant, and the images of MRI voxel-based morphometry demonstrated atrophy of the cerebellar cortex in the same area. Although the diagnosis was difficult to establish, cortical cerebellar atrophy was suspected. In addition, autoimmune cerebellar ataxia was suspected based on positive gliadin and thyroid antibodies and therefore the treatment with IVIg and steroid therapy was performed, leading to amelioration of truncal ataxia. Eight years after disease onset, the patient died and an autopsy was performed. The autopsy showed that the number of Purkinje cells in the cerebellum was moderately decreased in a selective manner. Consequently, the cerebellum was found to be the location of the culprit lesion causing truncal ataxia in the patient, leading to a definitive diagnosis of cortical cerebellar atrophy.

Moreover, prior to death, the patient had positive anti-SS-A and anti-SS-B antibodies without drying symptoms and was not thus diagnosed as having Sjögren syndrome. However, infiltration of lymphocytes as well as a decrease in the number of acini in the submandibular gland was found at autopsy. This led to the diagnosis of Sjögren syndrome.

In this patient, autoimmune cerebellar ataxia was suspected, given the findings of positive IgG and IgA antibodies to gliadin, positive anti-SS-A and anti-SS-B antibodies, and positive thyroid antibody, as well as the previous history of Basedow disease. Consequently, the treatment with intravenous immunogloblin and steroid therapy was performed, resulting in amelioration of truncal ataxia. A large number of reports on autoimmune cerebellar ataxia have been accumulated, including gluten ataxia [1], anti-GAD-antibody-positive cerebellar ataxia [2-4], Hashimoto encephalopathy [5,6], Sjögren syndrome [7], and neuro-Behçet disease [8]. This patient was diagnosed as having autoimmune cerebellar ataxia close to gluten ataxia because the patient had simple cortical cerebellar atrophy. This diagnosis was also appropriate given the lack of autopsy findings indicative of hereditary spinocerebellar degeneration such as SCA3 and SCA6 and multiple system atrophy, and because several types of autoantibodies [9] were found that have been reported previously in association with autoimmune cerebellar ataxia, including anti-gliadin antibody.

Gluten ataxia patients are known to be positive for anti-gliadin antibody. Hadjibassiliou et al. reported that anti-gliadin antibody cross-reacts with epitopes on Purkinje cells from human and rat cerebellum, and in doing so strengthens the impact of circulating antibodies against cerebellar Purkinje cells [10]. Hadjibassiliou et al. also reported post-mortem findings from two patients with gluten ataxia. Examination of the central nervous system revealed patchy loss of Purkinje cells throughout the cerebellar cortex. The cerebellar white matter showed astrocytic gliosis, and a diffuse infiltrate of mainly T-lymphocytes. Substantial perivascular cuffing with inflammatory cells, mainly T lymphocytes with smaller numbers of B lymphocytes and macrophages, was present within the cerebellar white matter and the posterior columns of the spinal cord [11]. In addition, Mittelbronn et al. reported that examination of the cerebellum of a 68-year-old male with gluten ataxia showed an absence of B- or plasma cells but multiple CD8^+^, suggesting that there are prominent cytotoxic effects in the neuropathogenesis of gluten ataxia [12]. The pathological findings of gluten ataxia in these reports differ from our case in that they show infiltration of inflammatory cells in the cerebellum. As described above, the pathological findings reported previously with gluten ataxia include infiltration of inflammatory cells in the cerebellum, which was not observed in our patient.

Along with gluten ataxia, paraneoplastic cerebellar ataxia and anti-GAD-antibody-positive cerebellar ataxia cause autoimmune damage to Purkinje cells in the cerebellum. A large number of autopsied cases of paraneoplastic cerebellar ataxia have been reported, but often without findings of inflammatory reactions. In the pathological investigation of three patients complicated with paraneoplastic cerebellar ataxia and LEMS, Fukuda et al. reported that a loss of Purkinje cells in the cerebellum was evident but HE staining and immunostaining using CD3, CD4, CD19, and CD20 did not demonstrate infiltration of lymphocytes in the cerebellar cortex [13]. Ishida et al. have also reported in an autopsied case of anti-GAD-antibody-positive cerebellar ataxia that near-complete depression of the Purkinje cells was observed without infiltration of inflammatory cells [14].

As stated above, only a few autopsies of gluten ataxia have been previously reported, however in both cases, immune therapy was not confirmed to be effective. We considered the diagnosis of autoimmune cerebellar ataxia possible in this case for the following reasons: immune therapy was effective; anti-gliadin antibody, anti-thyroid antibody and anti-SS-A antibody that are suggested to be related to autoimmune cerebellar ataxia were present; the patient's history of Basedow's disease; and the definite diagnosis of Sjögren's syndrome on autopsy. In the event that immune cerebellar neural disorders are preceded by humoral immunity, the pathological finding of poor infiltration of inflammatory cells, as found in this case and in some paraneoplastic syndromes can be explained. In this case, the administering of immune therapies including 2 instances of high-dose immunoglobulin therapy and steroid therapy might have affected the pathological findings.

Although antemortem diagnosis was difficult in the patient's life time, the loss of Purkinje cells that was found on autopsy led to the diagnosis of autoimmune cerebellar atrophy. Where this case differs from others that have been previously reported is in the atypical pathological finding of an absence of an inflammatory response. In the future we can expect an accumulation of autopsy cases and clarification of the corresponding pathological conditions, and this should provide more information on cases of autoimmune cerebellar ataxia, including gluten ataxia and anti-GAD-antibody-positive cerebellar ataxia.

## Competing interests

The authors declare that they have no competing interests.

## Consent

Written informed consent was obtained from a relative of the patient for publication of this case report and accompanying images. A copy of the written consent is available for review by the Editor-in-Chief of this journal.

## Authors' contributions

KN was a major contributor in writing the manuscript. MS participated in the pathological examination of the case. TT participated in collecting clinical data and images. AH and NT participated in its design and coordination and helped draft the manuscript. All authors read and approved the final manuscript.
